# Essentials of Pathology and Morbid Anatomy

**Published:** 1889-10

**Authors:** 


**Essentials of Pathology and Morbid Anatomy.** By C. E. Armand Semple, M. D., London. Philadelphia: W. B. Saunders. 1889. 159 pages; cloth, $1.00.

This compend is No. 6 of the Saunders's series of question compends published by this house, and in character and appearance is similar to the others of this series.

The advantages of compends, to aid the student of medicine in preparing for examination or otherwise, has been questioned. All compends are but the extracts of larger and complete works, and in a hurried review of a certain subject are valuable aids. In many cases, however, they lose their worth by enticing the student to peruse their pages, instead of attending at the lecture room, or to the careful study of standard text-books.

A compend of pathology and morbid anatomy labors under this last arraignment. Conciseness in describing pathological changes is to be striven after, but should not curtail that which is necessary in the elucidation of the subject under discussion.

This compend begins by taking up the discussion of the inflammations and inflammatory processes in the lungs, heart, blood-vessels, liver, kidney, stomach, brain, and spinal cord. These subjects are all treated, as far as possible, in a clear but concise manner, interspersed by several illustrations. Tuberculosis, the degenerations, and neoplasms are described more at length. The constitutional diseases and septic diseases are treated rather hurriedly, but adequately to outline their general pathological substrata.

The author dwells somewhat on the pathology of the urine; and a plate showing the various urinary deposits accompanies this chapter.

Under animal parasites are briefly described the various entozoa and dermatozoa, with good illustrations of trichina, the pediculi, and acari.

The closing chapter takes up the vegetable parasites. The classification of the different forms of bacteria and their characteristic mode of development makes this one of the most important and interesting of the whole book. Under the pathogenic bacilli are described the bacillus of anthrax, tuberculosis, leprosy, glanders, typhoid fever, cholera, etc. The cuts, showing these germs in their natural state, are very illustrative and nicely executed.

The typographical part of the work is very good, considering the low cost at which it is sold. W. C. K.

